# Childhood cognitive ability and body composition in adulthood

**DOI:** 10.1038/nutd.2016.30

**Published:** 2016-08-15

**Authors:** S M Kumpulainen, K Heinonen, M K Salonen, S Andersson, D Wolke, E Kajantie, J G Eriksson, K Raikkonen

**Affiliations:** 1 Institute of Behavioral Sciences, University of Helsinki, Helsinki, Finland; 2 National Institute for Health and Welfare, Helsinki, Finland; 3 Folkhälsan Research Unit, Helsinki, Finland; 4 Children's Hospital, Helsinki University Central Hospital and University of Helsinki, Helsinki, Finland; 5 Department of Psychology, University of Warwick, Coventry, UK; 6 Institute for Health and Welfare, Oulu, Finland; 7 Department of General Practice and Primary Health Care, University of Helsinki, Helsinki, Finland

## Abstract

**Background:**

Childhood cognitive ability has been identified as a novel risk factor for adulthood overweight and obesity as assessed by adult body mass index (BMI). BMI does not, however, distinguish fat-free and metabolically harmful fat tissue. Hence, we examined the associations between childhood cognitive abilities and body fat percentage (BF%) in young adulthood.

**Methods:**

Participants of the Arvo Ylppö Longitudinal Study (*n*=816) underwent tests of general reasoning, visuomotor integration, verbal competence and language comprehension (*M*=100; s.d.=15) at the age of 56 months. At the age of 25 years, they underwent a clinical examination, including measurements of BF% by the InBody 3.0 eight-polar tactile electrode system, weight and height from which BMI (kg m^−2^) was calculated and waist circumference (cm).

**Results:**

After adjustments for sex, age and BMI-for-age s.d. score at 56 months, lower general reasoning and visuomotor integration in childhood predicted higher BMI (kg m^−2^) increase per s.d. unit decrease in cognitive ability (−0.32, 95% confidence interval −0.60,−0.05; −0.45, −0.75,−0.14, respectively) and waist circumference (cm) increase per s.d. unit decrease in cognitive ability (−0.84, −1.56,−0.11; −1.07,−1.88,−0.26, respectively) in adulthood. In addition, lower visuomotor integration predicted higher BF% per s.d. unit decrease in cognitive ability (−0.62,−1.14,−0.09). Associations between general reasoning and BMI/waist were attenuated when adjusted for smoking, alcohol consumption, intake of fruits and vegetables and physical activity in adulthood, and all associations, except for visuomotor integration and BMI, were attenuated when adjusted for parental and/or own attained education and/or birth weight.

**Conclusions:**

Of the measured childhood cognitive abilities, only lower visuomotor integration was associated with BF% in adulthood. This challenges the view that cognitive ability, at least when measured in early childhood, poses a risk for adiposity in adulthood, as characterized by higher BF%.

## Background

Overweight (body mass index (BMI)⩾25 kg m^−2^) and obesity (BMI⩾30 kg m^−2^) are major global public health problems.^1, 2^ As their prevention and treatment are public health priorities, studies are urgently needed that attempt to identify, as early in life as possible, risk factors that may render individuals vulnerable to overweight and obesity and to their adverse physical and mental health consequences.

Research conducted over the past decade has identified childhood cognitive ability as a novel risk factor for obesity. These data come from a handful of studies showing that lower general intelligence measured at the age of 11 years was associated with higher BMI calculated from measured weight and height at the age of 70 years,^3^ and with overweight and obesity measured in follow ups conducted through ages from 79 to 90 years.^4^ Also one study demonstrated that lower verbal reasoning measured at the age of 3 years was associated with obesity measured at 38 years.^5^ In a series of other studies, lower general intelligence measured at 7–16 years was associated with higher BMI, overweight or obesity, as calculated from self-reported weight and height at ages from 30 to 52 years.^6, 7, 8, 9, 10^ We are aware of two studies reporting that childhood general intelligence measured at the age of 11 years was not associated with a gain in BMI when measured at the age of 16 years and when self-reported at age 42 years,^6^ or with BMI when measured in adulthood.^11^

While providing valuable information on the early life risk factors of overweight and obesity, all of the previous studies have relied solely on BMI. Further, only four of these studies have consistently calculated BMI from measured^3, 4, 5, 11^ rather than self-reported^6, 7, 8, 9, 10^ weight and height. The problem with BMI is that it does not distinguish between fat-free and fat tissue, although a higher amount of fat-free tissue, such as muscle, is associated with health benefits, whereas excess fat is harmful.^12^ The diagnostic performance of measured BMI to detect people with excess body fat percentage (BF%) is highly specific, but measured BMI has a poor sensitivity as it fails to identify nearly half of the people with excess BF%.^13^ Sensitivity and specificity of self-reported BMI to detect people with excess BF% are even poorer, as self-reports suffer from under-estimation bias.^14, 15^ Whether childhood lower cognitive abilities, including general reasoning, visuomotor integration, verbal competence and language comprehension, identifies individuals at risk for overweight and obesity characterized by higher BF% remains unknown.

Here we test the associations between general reasoning, visuomotor integration and verbal abilities at the age of 56 months and BF% measured at the age of 25 years using bioimpedance. These associations were tested, first, by using BF% as continuous, and, then, by testing whether non-obese and obese individuals, as identified by BF% cutoffs (⩾25% for men, ⩾35% for women) that are often used in epidemiological and clinical literature and recommended by the World Health Organization,^16, 17, 18, 19^ differed in childhood cognitive abilities. To allow comparisons with previous studies, we also report associations with BMI, and report associations with a proxy of central adiposity, namely waist circumference. Finally, because individuals classified as obese by BF% but non-obese by BMI have been shown to be at increased risk for cardio-metabolic diseases,^20, 21, 22^ we also tested whether individuals classified as non-obese and obese by either BF% or BMI or both differ from each other in childhood cognitive abilities.

## Methods

### Participants

Participants of the current study come from the Arvo Ylppö Longitudinal Study (AYLS) and the Finnish arm of the Bavarian Finnish Longitudinal Study.^23, 24^ They were recruited between 15 March 1985 and 14 March 1986 from the seven maternity hospitals in the county of Uusimaa. During the study period, 15 311 babies were born in the area, and of these 2193 (1193 boys) were recruited to the study. Of these babies, 1535 (867 boys) were admitted to the neonatal wards of the obstetric units, or transferred to the neonatal intensive care unit (NICU) of the Children's Hospital within 10 days of birth because the infant needed brief inpatient observation and treatment due to illnesses and complications. A large proportion of the hospitalized infants suffered from problems of a transient nature; hence, the majority of the admitted infants had no diagnosed illness and were on the ward for observation or because of common problems of neonatal adaptation.^23^ Of the babies, 658 (326 boys) were not hospitalized. Details of the study cohort are presented elsewhere.^23, 24, 25^

**Figure 1:**
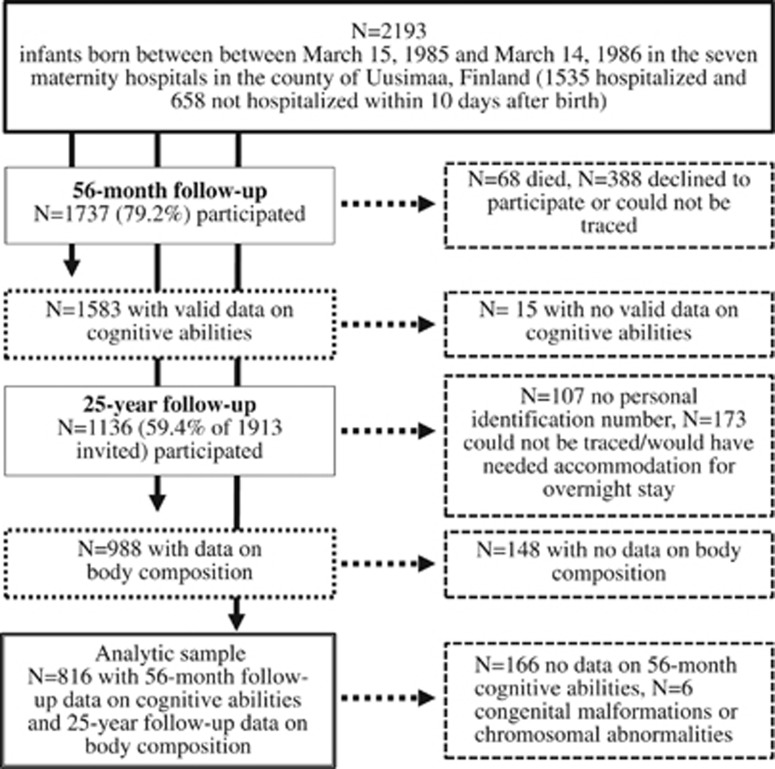
Flow chart of the number of participants of the Arvo Ylppö Longitudinal Study at follow-up visits conducted at 56 months and 25 years of age.

Figure 1 displays the participants of the study. In brief, of the 2193 babies in the original cohort, 1737 remained eligible for the follow up at 56 months (68 had died, 388 could not be traced). Of these, 1598 (75.2%) participated at an average age of 56 months (s.d.=0.46, range=54.74–61.84), and 1583 (858 boys) provided valid data on at least one of the tests for cognitive ability.

In 2009–2012, we invited the still traceable 1913 participants of the original cohort for a follow up (for 107 personal identification number was not available, for 173 addresses were not traceable, they were living abroad, or they would have needed accommodation for an overnight stay). Of those traceable, 1136 participated (59.4%) in the follow up at a mean age of 25.4 years (range=24.1–27.1) that included a measurement of body composition for 988 participants. Data on cognitive ability at the age of 56 months and body composition at the age of 25.4 years were available for 822 participants. After excluding six participants with congenital malformations or chromosomal abnormalities, the analytic sample of the current study comprised 816 individuals (409 women, 407 men).

Those in the analytic sample (*n*=816) and those who were invited but who did not participate (*n*=776) did not differ from each other in age, weight, height, BMI or language comprehension at the age of 56 months (all *P*-values >0.05); the analytic sample included more frequently women (50.1% vs 41.2%, *P*<0.001), weighed more at birth (mean difference (MD) in s.d. units of birth weight for sex=0.13, *P*<0.01), had more frequently parent(s) with upper tertiary education (32.1% vs 24.0%, *P*<0.001), and scored higher on general reasoning (MD=5.06, *P*<0.001), visuomotor integration (MD=5.18, *P*<0.001) and verbal competence (MD=3.55, *P*<0.001) in childhood. The analytic sample also included more frequently participants who were not hospitalized after birth (35.8% vs 27.7%, *P*<0.001). We have previously reported that the hospitalized and non-hospitalized participants did not differ in physical growth up to 56 months or in childhood cognitive abilities.^23^ They also did not differ in BF% or BMI (*P*-values >0.065), but had a smaller waist circumference (MD=2.29, *P*<0.05) in adulthood.

The childhood protocol was approved by the Ethics Committees of Helsinki City Maternity Hospital, Helsinki University Central Hospital and Jorvi Hospital, and in adulthood by the Coordinating Ethics Committee of the Helsinki and Uusimaa Hospital District. An informed consent was obtained from parents (childhood) and participants (adulthood).

### Measures

#### Cognitive abilities at 56 months

The test battery comprised four valid and reliable tests that are used to screen for individual differences in cognitive abilities,^25, 26, 27, 28, 29, 30, 31, 32, 33^ but none alone can yield a clinical diagnosis of intellectual disability. First, non-verbal general reasoning ability was measured using the Columbia Mental Maturity Scale for 3- to 10-year-olds.^34^ It consists of 100 cards displaying a set of 3–5 drawings from which the child is asked to select the one that is different from or unrelated to the others. A sum score of correct choices ranges from 0 to 100.

Second, ability to integrate visual and motor abilities was measured using the Beery Scale for 3- to 15-year-old children.^28^ The child is asked to copy 12 geometric figures that are presented in order of increasing difficulty. Each drawing is evaluated to be either correct or incorrect by standardized norms, and the sum score ranges from 0 to 15.

Third, verbal competence (expressive vocabulary) was measured using a Finnish translation of the test by Kiese and Kozielski^35^ for 3- to 5-year-olds. It comprises 82 picture-naming tasks, being similar to the Peabody picture vocabulary test of verbal intelligence. A sum score of correct choices ranges from 0 to 82.

Fourth, language comprehension was assessed using the Finnish Logopädisher Sprachverständnis Test.^36, 37^ In this study, we used part A of the test for 4- to 8-year-old children, comprising a set of standard toys with which the child is asked to follow the actions verbally requested by the examiner.^37^ A sum score of correctly executed tasks ranges from 0 to 17.

All of the cognitive test scores were corrected for exact age at measurement and converted to usual intelligence-type scores with a mean of 100 and s.d. of 15 (see, for example, Hart *et al.*^11^).

#### Body composition, body mass index and waist circumference at 25 years of age

Body composition was measured using bioelectrical impedance by the InBody 3.0 eight-polar tactile electrode system (Biospace Co. Ltd, Seoul, Korea). This instrument estimates BF% by segmental multifrequency (5, 50, 25 and 500 kHz) analyses separately for each limb and trunk. To identify obesity by BF%, we used cutoffs of ⩾25% for men and ⩾35% for women.^16, 17, 18, 19^

In addition, weight (kg) and height (cm) were measured in light clothes without shoes and BMI (kg m^−2^) was calculated. Waist circumference (cm) was measured twice midway between the lowest rib and the iliac crest, and the mean of these two measurements was recorded.

### Covariates and confounders

These included sex (men/women), age (years) in adulthood and BMI (kg m^−2^) calculated from weight and height measured in a clinic and transformed into BMI-for-age s.d. scores using national growth references for girls and boys,^38^ self-reported lifestyle and dietary factors in adulthood, including alcohol consumption (g per week), smoking status (yes, ex-smoker and no), and leisure-time conditioning physical activity (I do tasks that do not demand much movement and do not cause physical strain; I perform exercise not causing substantial perspiration at least 4 h per week; I exercise to maintain my physical condition for at least 3 h per week; and I compete or train several times per week), average daily intakes of energy (kcal per day), fruits and vegetables (g per day),^39^ mother-reported parental education (highest of either parent) at the 56-month visit, own education in adulthood as self-reported (primary/elementary and middle school, secondary/high school, lower tertiary/college and upper tertiary/university) and birth weight (g) standardized by sex derived from hospital birth records. All categorical variables were dummy coded. Previous studies have shown that these covariates and confounders are related to adiposity and/or cognitive ability.^3, 4, 6, 7, 8, 9, 10, 11^

### Statistical analysis

By using multiple linear regression analysis, we examined whether general reasoning and visuomotor and verbal abilities at 56 months of age were associated with BF%, BMI and waist circumference in adulthood. In addition, logistic regression analysis was used to test these associations when BF% was dichotomized at the obesity cutoff. Data met the assumptions of the test used.

We also tested whether individuals with BMI and BF% at or above and below the obesity cutoffs differed from each other in childhood cognitive abilities by using univariate analysis of covariance.

We report the associations first as adjusted for sex, age in adulthood and BMI-for-age s.d. score at 56 months (model 1); plus lifestyle and dietary factors in adulthood (model 2); plus parental education at 56 months of age (model 3); plus own attained education in adulthood (model 4); plus birth weight standardized by sex (model 5).

We also tested whether the associations were non-linear by including a cognitive ability squared term in the regression equation. This model also included cognitive ability linear term plus model 1 covariates.

## Results

Table 1 shows that compared with individuals in the non-obese BF% group (<25% for men,<35% for women), those in the obese BF% group (⩾25% for men, ⩾35% for women) had higher weight and BMI at 56 months, higher weight, BMI, and waist circumference and shorter stature at 25 years, more often had parents with lower levels of education, and had themselves by the age of 25 years achieved lower and less often higher levels of education, and reported lower intake of fruits and vegetables, higher levels of alcohol consumption, and more often lower levels and less often higher levels of leisure time physical activity at the age of 25 years. The groups did not differ from each other in the other covariates/confounders.

Of the childhood cognitive abilities, only higher verbal competence correlated with higher childhood BMI (*r*=0.085, *P*=0.019; *P*-values >0.090 for correlations of general reasoning, visuomotor integration and language comprehension with childhood BMI).

### Associations between cognitive abilities and BF%, BMI and waist circumference

Associations between cognitive abilities at 56 months of age and BF%, BMI and waist circumference at 25 years of age are presented in Table 2. After adjusting for sex, age in adulthood and BMI s.d. score at 56 months (model 1), BF% in adulthood was higher by 0.62% for each s.d. unit decrease in childhood visuomotor integration. Logistic regression analyses showed a similar finding; for each s.d. unit decrease in visuomotor integration, the change in odds for belonging to the group with higher BF% (⩾25% for men and ⩾35% for women) increased by 30% (odds ratio=1.30, 95% confidence interval: 1.05, 1.62, *P*=0.02). This association remained significant in the linear (Table 2) and logistic regression analyses (odds ratio=1.34, 95% confidence interval: 1.05, 1.70, *P*=0.02) when we made further adjustments for adulthood lifestyle/dietary factors, but when we made adjustments for parental or own attained level of education and for birth weight, it was rendered non-significant (Table 2; *P*-values for logistic regression analyses results **>**0.054, data not shown). General reasoning, verbal competence and language comprehension were not significantly associated with BF% in either linear (Table 2) or logistic regression analyses (*P*-values >0.18, data not shown). None of the associations were non-linear (Table 2).

BMI in adulthood increased by 0.32 and 0.45 kg m^−2^ and waist circumference by 0.84 and 1.1 cm for each s.d. unit decrease in childhood general reasoning and visuomotor integration, respectively (Table 2). These associations held after all model adjustments, except when adjusted for lifestyle/dietary factors and birth weight, the associations between general reasoning and BMI and waist circumference were rendered non-significant (Table 2). When we made further adjustment for parental or own attained level of education, the associations between general reasoning and BMI, between general reasoning and waist circumference, between visuomotor integration and BMI and between visuomotor integration and waist circumference were rendered non-significant (Table 2). Verbal competence and language comprehension were not significantly associated with BMI or waist circumference (Table 2). None of these associations were non-linear (Table 2).

Results from analyses testing differences in childhood cognitive abilities in individuals classified as obese and non-obese by BMI and BF% showed that the groups did not differ significantly from each other (*P*-values >0.059; data not shown).

The associations between cognitive abilities at 56 months of age and body fat mass (kg) and fat-free mass (kg) at the age of 25 years are presented in Supplementary Table 1. Lower visuomotor integration was associated with higher fat mass (*P*-values varied from 0.009 to 0.08 in models 1–5) and general reasoning showed a non-linear association with fat-free mass such that those with lower and higher general reasoning had the lowest fat-free mass values (*P*=0.021). This non-linear association held in all adjustment models (*P*-values <0.029; data not shown).

### Exploratory/secondary analyses

Given that only visuomotor integration at the age of 56 months was associated with BF%, we conducted exploratory analyses to examine whether associations between childhood cognitive abilities and BF% in adulthood were moderated by sex, BMI-for-age s.d. score at 56 months, parental level of education in childhood, own attained level of education in adulthood and birth weight. ‘Birth weight × general reasoning' (*P*<0.001), ‘birth weight × verbal competence' (*P*=0.029) and ‘BMI-for-age s.d. score at 56 months × general reasoning' (*P*<0.01) interactions were significant in the analyses of BF%. For further exploration, we formed dichotomous groups according to birth weight (<2.5 vs ⩾2.5 kg) and BMI-for-age s.d. score (median split <−0.30 vs ⩾−0.30). However, in neither birth weight group and in neither BMI group were the associations between cognitive abilities and BF% significant (*P*-values >0.056; data not shown).

We also examined whether associations between childhood cognitive abilities and BF% in adulthood were moderated by hospitalization within 10 days of birth. None of the ‘hospitalization × childhood cognitive ability' interactions were significant (*P*-values >0.09).

### BF% and BMI: sensitivity and specificity

Finally, because BF% and BMI yielded somewhat different results, we calculated the sensitivity and specificity of BMI to detect excess adiposity in this sample.

BMI cutoff of ⩾30 kg m^−2^ yielded a sensitivity of 0.61 and a specificity of 0.98 to detect excess adiposity (⩾25% for men, ⩾35% for women; sensitivity of 0.66 and 0.57 and specificity of 0.97 and 0.99 for men and women, respectively) (Supplementary Figure 2).

## Discussion

To our knowledge, this is the first study to examine the associations between childhood general reasoning and visuomotor and verbal abilities with adulthood body composition as defined by BF% in a large sample of women and men followed up from birth to 25 years of age. Contrary to previous findings using BMI, our results indicate that of the cognitive abilities tested childhood general reasoning and verbal ability were not associated with adulthood BF%, and that only lower visuomotor integration in childhood was associated with higher adulthood BF%. The latter association was significant when we made adjustments for sex, adult age, and childhood BMI and also remained significant when we made further adjustments for adult smoking, alcohol consumption, intake of fruits and vegetables and physical activity, but when we made adjustments for parental level of education in childhood, own attained level of education in adulthood and birth weight, this association was rendered non-significant. We did not find differences in general, visuomotor or verbal childhood cognitive abilities when we classified individuals as obese and non-obese based on both BF% and BMI. These findings thus suggest that when adiposity is defined by BF% instead of BMI in adulthood, only visuomotor integration is associated with adult adiposity. Our findings therefore challenge the view that lower childhood cognitive abilities, at least when measured in early childhood, pose a risk for adiposity characterized by excess BF% in adulthood.

However, when we used BMI (or waist circumference) instead of BF% as an index of adulthood adiposity, we found an association between lower childhood general reasoning and lower visuomotor integration with higher BMI (or waist circumference) in adulthood. This finding is, indeed, in line with the previous cognitive epidemiological studies^3, 4, 5, 6, 7, 8, 9, 10^ as well as our study hypothesis suggesting that lower childhood general and visuomotor abilities pose a risk for adulthood overweight and obesity. Also in line with the previous studies,^7, 8, 9^ own attained education weakened and parental educational level further attenuated these associations to non-significance. It has been suggested that this may simply reflect overadjustment and/or mediation via education, as childhood parental and own attained education in adulthood are close correlates of cognitive ability.^40, 41^ Indeed, a number of studies have consistently demonstrated that low level of education is associated with obesity and poorer physical and mental health.^42^

The discrepancies in the associations when using BF% and BMI reflect their differences in detecting adiposity. Although BMI has been widely used in epidemiological studies, its limitations in diagnosing excess adiposity have been recognized.^43^ BMI also fails to distinguish between fat-free and fat mass, particularly when BMI values are below 30 kg m^−2^. The group with intermediate BMI values may include those who have higher levels of muscle mass and less fat mass (for example, athletes) and those who have high levels of fat mass and less fat-free mass (that is, ‘normal-weight obese').^13^ A meta-analysis of a total of 31 968 individuals suggests that BMI results in underdiagnosing excess body adiposity in nearly half of individuals.^13^ Sensitivity and specificity calculated in this sample were in line with the findings of this meta-analysis, as BMI in our sample performed well in detecting excess body adiposity, but nearly 40% of those who were not obese according to BMI had excess body adiposity according to BF%, and 2% who were obese by BMI were not obese by BF%. Further, the importance of measuring BF% instead of BMI comes from studies that have shown that individuals who have BMI within the normal range but who have high BF% have an increased risk for cardio-metabolic diseases.^20, 21, 22^ Of note is that when we tested associations separately with fat mass and fat-free mass, we detected associations with fat mass, but no consistent associations with fat-free mass.

In interpreting our findings, one should keep in mind that our participants were fairly young when their cognitive abilities were tested and still relatively young at measurement of BF% in adulthood. While cognitive abilities have a relatively high rank-order stability from childhood to adulthood,^44, 45^ it is likely that they have not reached their full potential at the age of 56 months, and that, for instance, entering school, which in Finland takes place at the age of 7 years, might have an effect on cognitive ability test results. Education may especially impact verbal abilities, the abilities of which were not associated with any later outcomes in our study. Further, while obesity tends to be stable from adolescence to young adulthood,^46^ and to midlife,^47^ its prevalence increases with age^48^ and peaks in midlife.^1^ Hence, variance in adiposity in our young adult sample may have been narrower than in the previous studies on middle-aged and older populations. However, our sample comprised 186 (22.8%) overweight and 88 (10.8%) obese individuals according to BMI cutoffs, and 125 obese individuals (15.3%) according to the BF% cutoff.

Strengths of our study include the relatively large sample size, the long follow up, measurement of BF% by bioelectrical impedance and availability of data on measured BMI and waist circumference, which enabled comparisons with previous studies. Some limitations exist as well. While multi-frequency bioelectrical impedance is a practical and non-invasive method to measure BF% in a large epidemiological setting, it may overestimate BF% in the obese BMI group.^49^ Although the results concerning the validity of multi-frequency bioelectrical impedance vary between studies,^49, 50, 51^ the reliability of multi-frequency bioelectrical impedance is shown to be excellent in standardized conditions. Further studies using more precise estimation methods, such as dual-energy X-ray absorptiometry and under-water weighing, are clearly warranted. Also, no consensus exists on the BF% cutoffs to identify excess body fat. However, our results did not differ when we used the BF% cutoffs and when we used BF% as continuous in our analyses.

Two-thirds of the infants participating in the AYLS were admitted to the neonatal ward after birth. However, the majority of them had no diagnosed illness and were on the ward for observation or because of common transient problems in neonatal adaptation. An indication of this is that there were no differences in somatic growth up to 56 months or in cognitive abilities between the admitted and the non-admitted infants.^23^ Further, in the current study, cognitive abilities were assessed at only one point in childhood. Repeated assessments would have enabled us to test whether change in cognitive ability and abilities at different age stages had different effects on adulthood adiposity. Yet, one recent study has demonstrated that obesity in adulthood was not associated with change in intelligence from childhood to adulthood.^5^ We also lacked data on childhood BF%. Repeated assessments of BF% and cognitive ability in childhood would have enabled us to examine whether any associations between body composition and cognitive ability were bidirectional. Finally, in longitudinal studies, loss to follow up is inevitable and may cause a potential selection bias. Participants in the current study had better educated parents, higher scores on general reasoning, visuomotor integration and verbal competence in childhood, were more often women and non-hospitalized controls, and weighed more at birth. Hence, our findings may not generalize to populations differing in these characteristics from our sample.

In conclusion, our study showed that of the measured childhood cognitive abilities only visuomotor ability was associated with BF% in adulthood. This challenges the view that lower cognitive abilities, at least when measured in early childhood, pose a risk for adiposity defined by higher BF% in adulthood.

## Figures and Tables

**Table 1:** Characteristics of the sample according to body fat percentage (BF%)

	*BF%*	*BF%*	*Group difference* P
	*Men<25% Women<35%* n=*691*	*Men*⩾*25% Women*⩾*35%* n=*125*	
	*Mean (s.d.)/*n *(%)*	*Mean (s.d.)/*n *(%)*	
*At birth*			
Sex (boys)	349 (50.5%)	58 (46.4%)	0.398
Gestation length (weeks)	39.0 (2.68)	38.6 (3.08)	0.182
Birth weight (grams)	3349.9 (737.1)	3312.5 (208.8)	0.608
Birth weight (s.d. units by sex)	0.13 (0.91)	0.09 (1.01)	0.711
			
*At 56-month follow up*			
Age (months)	56.5 (0.4)	56.5 (0.4)	0.660
Weight (kg)	17.97 (2.4)	19.3 (3.41)	0.000
Height (cm)	108.2 (4.4)	108.5 (4.9)	0.398
Body mass index (kg m^−2^)	15.3 (1.3)	16.3 (1.9)	0.000
Body mass index-for-age (s.d. units)	−0.5 (0.9)	0.2 (1.3)	0.000
Highest education of either parent			0.007
Basic/primary or less (elementary and middle school)	55 (8.0%)	9 (7.2%)	
Upper secondary (high school)	145 (21.0%)	39 (31.2%)	
Lower tertiary (college)	255 (36.9%)	52 (41.6%)	
Upper tertiary (university)	236 (34.2%)	25 (20.0%)	
*Cognitive ability (*M=*100; s.d.=15)*			
General reasoning	100.0 (16.7)	98.7 (16.2)	0.407
Visuomotor integration	101.0 (14.7)	98.1 (14.4)	0.042
Verbal competence	100.4 (14.5)	101.3 (14.7)	0.550
Language comprehension	99.7 (14.4)	101.5 (15.8)	0.214
			
*At 25-year follow up*			
Age (years)	25.4 (0.6)	25.3 (0.6)	0.847
Weight (kg)	68.9 (12.9)	93.8 (17.4)	0.000
Height (cm)	173.2 (9.3)	171.4 (9.7)	0.042
Body mass index (kg m^−2^)	22.8 (3.0)	31.8 (4.6)	0.000
⩾30	12 (1.7%)	76 (60.8%)	
25.00–29.99	141 (20.4%)	45 (36.0%)	
18.50–24.99	502 (72.6%)	4 (3.2%)	
<18.5	36 (5.2%)	0 (0.0%)	0.000
Waist circumference (cm)	79.7 (9.3)	102.1 (12.3)	0.000
Men⩾102 cm, women⩾88 cm	30 (4.3%)	95 (76.0%)	0.000
Level of education			0.006
Basic/primary or less (elementary and middle school)	31 (4.6%)	12 (10.1%)	
Upper secondary (high school)	205 (30.4%)	51 (42.9%)	
Lower tertiary (college)	194 (28.7%)	33 (27.7%)	
Upper tertiary (university)	245 (36.3%)	23 (19.3%)	
Alcohol consumption (g per week)	69.8 (85.1)	93.4 (161.8)	0.021
Fruit and vegetable consumption (g per day)	465.9 (290.3)	386.7 (293.4)	0.006
Energy intake (kcal per day)	2246.6 (791.5)	2123.1 (80.5)	0.119
Cigarette smoking			0.253
Non-smoker	151 (22.3%)	29 (24.0%)	
Ex-smoker	265 (39.2%)	38 (31.4%)	
Smoker	260 (38.5%)	54 (44.6%)	
Leisure time physical activity			0.000
Not much	141 (21.9%)	49 (43.0%)	
Without substantial perspiration at least 4 h per week	169 (26.2%)	40 (35.1%)	
Conditioning at least 3 h per week	279 (43.3%)	21 (18.4%)	
Competing, training several times per week	56 (8.7%)	4 (3.5%)	

**Table 2:** Associations between cognitive ability at the age of 56 months and body fat percentage, body mass index and waist circumference at the age of 25 years

*Cognitive ability*	*Body fat percentage*			*Body mass index (kg m*^−*2*^)			*Waist circumference (cm)*		
	*Unstandardized regression coefficient*^a^	*95% confidence interval*	P*-value*	*Unstandardized regression coefficient*^a^	*95% confidence interval*	P*-value*	*Unstandardized regression coefficient*^a^	*95% confidence interval*	P*-value*
*General reasoning (s.d. units)*									
Model 1	−0.24	(−0.71, 0.23)	0.313	−0.32	(−0.60, −0.05)	0.024	−0.84	(−1.56, −0.11)	0.024
Model 2	−0.29	(−0.77, 0.21)	0.259	−0.27	(−0.57, 0.03)	0.074	−0.71	(−1.47, 0.06)	0.068
Model 3	−0.02	(−0.50, 0.47)	0.966	−0.20	(−0.48, 0.09)	0.174	−0.53	(−1.26, 0.23)	0.170
Model 4	−0.11	(−0.59, 0.38)	0.668	−0.21	(−0.50, 0.06)	0.136	−0.53	(−1.26, 0.21)	0.159
Model 5	−0.14	(−0.62, 0.33)	0.563	−0.27	(−0.56, 0.00)	0.054	−0.72	(−1.46, 0.02)	0.053
Model 6	0.00	(−0.02, 0.02)	0.614	0.00	(−0.02, 0.00)	0.473	−0.02	(−0.05, 0.02)	0.293
									
*Visuomotor integration (s.d. units)*									
Model 1	−0.62	(−1.14, −0.09)	0.021	−0.45	(−0.75, −0.14)	0.005	−1.07	(−1.88, −0.26)	0.010
Model 2	−0.63	(−1.16, −0.11)	0.020	−0.42	(−0.75, −0.09)	0.012	−0.93	(−1.76, −0.11)	0.027
Model 3	−0.39	(−0.93, 0.15)	0.153	−0.32	(−0.63, 0.02)	0.056	−0.72	(−1.56, 0.12)	0.094
Model 4	−0.47	(−1.01, 0.06)	0.085	−0.32	(−0.63, 0.00)	0.049	−0.69	(−1.52, 0.12)	0.096
Model 5	−0.51	(−1.04, 0.03)	0.064	−0.41	(−0.72, −0.09)	0.012	−0.96	(−1.79, −0.14)	0.023
Model 6	0.00	(−0.03, 0.03)	0.858	0.00	(−0.02, 0.02)	0.784	0.02	(−0.05, 0.06)	0.735
									
*Verbal competence (s.d. units)*									
Model 1	0.12	(−0.42, 0.66)	0.654	0.00	(−0.32, 0.33)	0.997	0.12	(−0.72, 0.96)	0.772
Model 2	0.23	(−0.35, 0.80)	0.443	0.09	(−0.26, 0.45)	0.590	0.41	(−0.48, 1.29)	0.372
Model 3	0.36	(−0.20, 0.92)	0.210	0.15	(−0.18, 0.50)	0.354	0.50	(−0.36, 1.35)	0.252
Model 4	0.29	(−0.27, 0.83)	0.318	0.14	(−0.20, 0.45)	0.442	0.50	(−0.36, 1.34)	0.253
Model 5	0.20	(−0.35, 0.74)	0.475	0.05	(−0.29, 0.36)	0.805	0.23	(−0.62, 1.07)	0.606
Model 6	0.00	(−0.03, 0.03)	0.812	0.00	(−0.02, 0.02)	0.754	0.02	(−0.03, 0.06)	0.479
									
*Language comprehension (s.d. units)*									
Model 1	0.00	(−0.54, 0.53)	0.995	−0.06	(−0.38, 0.26)	0.706	−0.09	(−0.92, 0.74)	0.830
Model 2	0.03	(−0.51, 0.59)	0.908	−0.03	(−0.36, 0.32)	0.887	0.03	(−0.83, 0.87)	0.954
Model 3	0.09	(−0.45, 0.62)	0.752	−0.02	(−0.33, 0.30)	0.943	0.05	(−0.78, 0.87)	0.909
Model 4	0.11	(−0.44, 0.63)	0.707	0.03	(−0.29, 0.35)	0.882	0.17	(−0.66, 0.98)	0.706
Model 5	0.03	(−0.51, 0.56)	0.931	−0.06	(−0.38, 0.27)	0.740	−0.06	(−0.90, 0.77)	0.875
Model 6	0.02	(0.00, 0.05)	0.090	0.02	(0.00, 0.03)	0.059	0.03	(−0.02, 0.06)	0.169

a Negative coefficient indicates that lower cognitive ability is associated with higher adiposity and positive coefficient that higher cognitive ability is associated with higher adiposity; s.d. units refer to standard deviation units with mean of 100 and s.d. of 15. Model 1 refers to adjustment for sex, age in adulthood and body mass index-for-age s.d. score at 56 months; Model 2 refers to model 1 covariates/confounders plus lifestyle/dietary factors (smoking, alcohol consumption, physical activity and intake of fruits and vegetables) in adulthood; Model 3 refers to model 1 covariates/confounders plus parental education at 56 months; Model 4 refers to model 1 covariates/confounders plus own attained education in adulthood; Model 5 refers to model 1 covariates/confounders plus birth weight standardized by sex; Non-linear refers to a model including cognitive ability squared term in a model that also includes cognitive ability linear term and model 1 covariates/confounders.
