# Supplementary material for: Childhood cognitive ability and body composition in adulthood
Source: Nutr Diabetes. 2016 Aug 15;6(8):e223–. doi: 10.1038/nutd.2016.30 (PMC5022144; doi:10.1038/nutd.2016.30)
Supplement: Supplementary Figure 1 Legend [file nutd201630x1.docx]

Supplementary Figure 1. Receiver operating characteristic (ROC) curve for body mass index (BMI) ≥30 kg/ m^2^ to detect excess body fat, area under curve is 0.97, 95% Confidence Interval is 0.95, 0.98.
