# Supplementary figures and images for: Childhood cognitive ability and body composition in adulthood
Source: Nutr Diabetes. 2016 Aug 15;6(8):e223–. doi: 10.1038/nutd.2016.30 (PMC5022144; doi:10.1038/nutd.2016.30)

## Slide 1
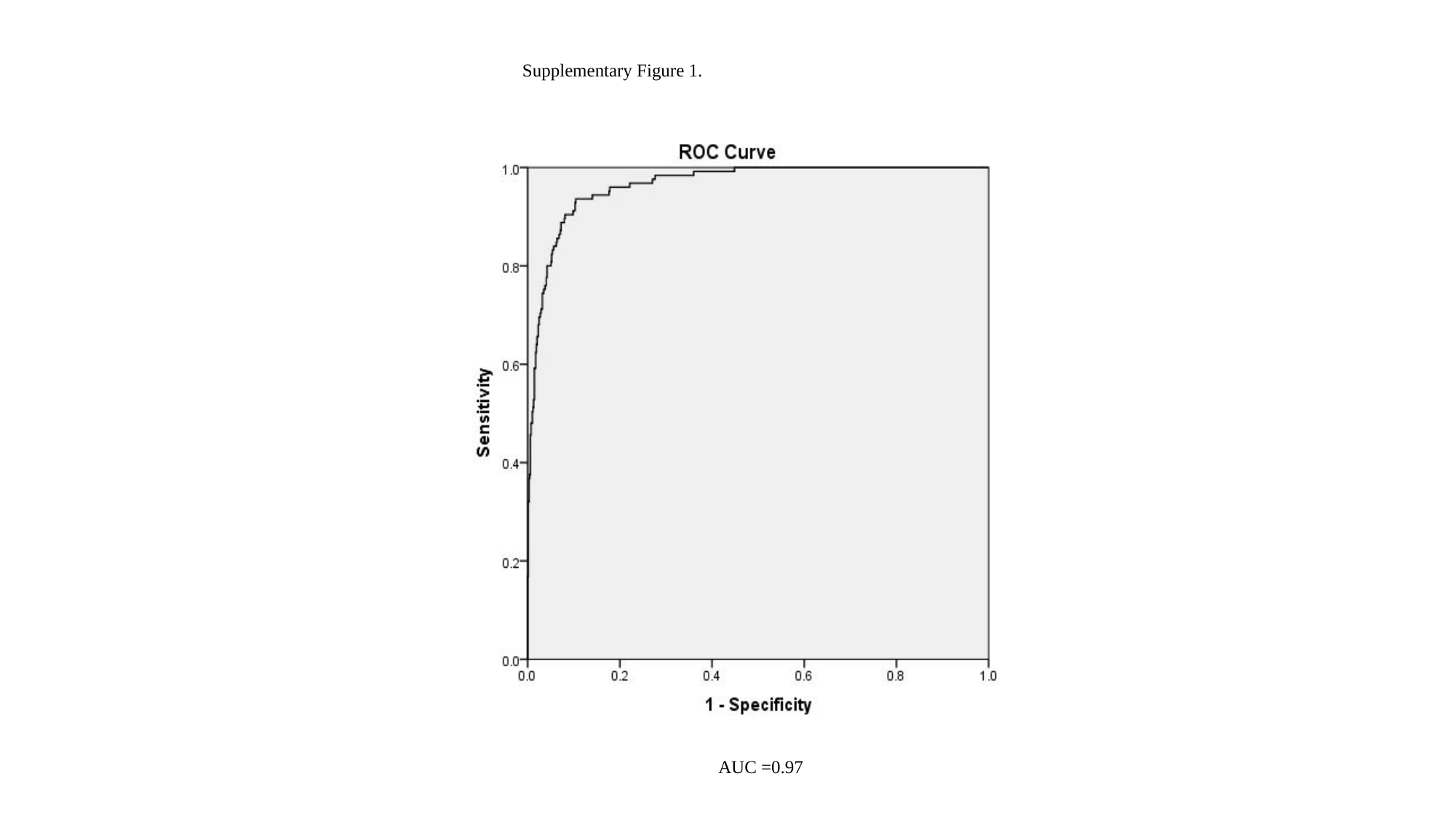

Supplementary Figure 1.
AUC =0.97

Supplement: Supplementary Figure 1 [file nutd201630x2.ppt]
