# Supplementary material for: Childhood cognitive ability and body composition in adulthood
Source: Nutr Diabetes. 2016 Aug 15;6(8):e223–. doi: 10.1038/nutd.2016.30 (PMC5022144; doi:10.1038/nutd.2016.30)
Supplement: Supplementary Table 1 [file nutd201630x3.docx]

| **Supplementary Table 1. Associations between cognitive ability at the age of 56 months and body fat mass and fat-free mass at the age of 25 years.** | | | | | | |
| --- | --- | --- | --- | --- | --- | --- |
|  | **Body fat mass (kg)** | | | **Fat-free mass (kg)** | | |
| **Cognitive ability:** | Unstandardized regression coefficient* | 95% confidence interval | P | Unstandardized regression coefficient* | 95% confidence interval | P |
| **General Reasoning (SD units)** | | | | | | |
| Model 1 | -0.50 | (-1.08, 0.08) | 0.088 | -0.24 | (-0.69, 0.21) | 0.302 |
| Model 2 | -0.53 | (-1.13, 0.09) | 0.092 | -0.14 | (-0.62, 0.36) | 0.602 |
| Model 3 | -0.24 | (-0.83, 0.35) | 0.427 | -0.18 | (-0.66, 0.29) | 0.440 |
| Model 4 | -0.30 | (-0.89, 0.29) | 0.315 | -0.18 | (-0.65, 0.29) | 0.452 |
| Model 5 | -0.44 | (-1.02, 0.15) | 0.147 | -0.44 | (-0.89, 0.02) | 0.059 |
| Model 6 | -0.02 | (-0.03, 0.02) | 0.407 | -0.02 | (-0.03, 0.00) | 0.021 |
| **Visuomotor Integration (SD units)** | | | | | | |
| Model 1 | -0.86 | (-1.50, -0.21) | 0.009 | -0.09 | (-0.62, 0.42) | 0.723 |
| Model 2 | -0.87 | (-1.52, -0.21) | 0.010 | -0.02 | (-0.57, 0.53) | 0.945 |
| Model 3 | -0.59 | (-1.25, 0.08) | 0.083 | -0.02 | (-0.56, 0.53) | 0.942 |
| Model 4 | -0.63 | (-1.28, 0.03) | 0.060 | 0.00 | (-0.53, 0.53) | 0.994 |
| Model 5 | -0.78 | (-1.44, -0.14) | 0.019 | -0.36 | (-0.89, 0.15) | 0.164 |
| Model 6 | 0.00 | (-0.03, 0.05) | 0.956 | -0.02 | (-0.05, 0.02) | 0.199 |
| **Verbal Competence (SD units)** | | | | | | |
| Model 1 | 0.20 | (-0.47, 0.86) | 0.571 | 0.20 | (-0.33, 0.74) | 0.458 |
| Model 2 | 0.32 | (-0.39, 1.02) | 0.384 | 0.38 | (-0.21, 0.96) | 0.202 |
| Model 3 | 0.50 | (-0.18, 1.17) | 0.154 | 0.29 | (-0.27, 0.83) | 0.315 |
| Model 4 | 0.44 | (-0.24, 1.10) | 0.211 | 0.30 | (-0.26, 0.84) | 0.289 |
| Model 5 | 0.26 | (-0.41, 0.92) | 0.457 | 0.11 | (-0.42, 0.63) | 0.699 |
| Model 6 | 0.00 | (-0.03, 0.03) | 0.864 | 0.00 | (-0.02, 0.03) | 0.590 |
| **Language Comprehension (SD units)** | | | | | | |
| Model 1 | -0.12 | (-0.77, 0.54) | 0.731 | 0.14 | (-0.39, 0.66) | 0.617 |
| Model 2 | -0.06 | (-0.74, 0.63) | 0.876 | 0.29 | (-0.27, 0.84) | 0.313 |
| Model 3 | -0.02 | (-0.66, 0.65) | 0.975 | 0.15 | (-0.38, 0.68) | 0.564 |
| Model 4 | 0.05 | (-0.62, 0.69) | 0.902 | 0.20 | (-0.33, 0.72) | 0.476 |
| Model 5 | -0.11 | (-0.77, 0.56) | 0.758 | 0.05 | (-0.47, 0.57) | 0.851 |
| Model 6 | 0.03 | (0.00, 0.06) | 0.045 | 0.02 | (-0.02, 0.03) | 0.399 |
| Note: *negative coefficient indicates that lower cognitive ability is associated with higher adiposity and positive coefficient that higher cognitive ability is associated with higher adiposity; SD units refer to standard deviation units with mean of 100 and standard deviation of 15. Model 1 refers to adjustment for sex, age in adulthood, and body mass index-for-age SD score at 56 months; Model 2 refers to model 1 covariates/confounders plus lifestyle/dietary factors (smoking, alcohol consumption, physical activity, and intake of fruits and vegetables) in adulthood; Model 3 refers to model 1 covariates/confounders plus parental education at 56 months; Model 4 refers to model 1 covariates/confounders plus own attained education in adulthood; Model 5 refers to model 1 covariates/confounders plus birth weight standardized by sex; Non-linear refers to a model including cognitive ability squared term in a model that also includes cognitive ability linear term and model 1 covariates/confounders. | | | | | | |
